# Embodied Evolution in Collective Robotics: A Review

**DOI:** 10.3389/frobt.2018.00012

**Published:** 2018-02-22

**Authors:** Nicolas Bredeche, Evert Haasdijk, Abraham Prieto

**Affiliations:** ^1^Sorbonne Université, CNRS, Institute of Intelligent Systems and Robotics, ISIR, Paris, France; ^2^Computational Intelligence Group, Department of Computer Science, Vrije Universiteit, Amsterdam, Netherlands; ^3^Integrated Group for Engineering Research, Universidade da Coruna, Ferrol, Spain

**Keywords:** embodied evolution, online distributed evolution, collective robotics, evolutionary robotics, collective adaptive systems

## Abstract

This article provides an overview of evolutionary robotics techniques applied to online distributed evolution for robot collectives, namely, embodied evolution. It provides a definition of embodied evolution as well as a thorough description of the underlying concepts and mechanisms. This article also presents a comprehensive summary of research published in the field since its inception around the year 2000, providing various perspectives to identify the major trends. In particular, we identify a shift from considering embodied evolution as a parallel search method within small robot collectives (fewer than 10 robots) to embodied evolution as an online distributed learning method for designing collective behaviors in swarm-like collectives. This article concludes with a discussion of applications and open questions, providing a milestone for past and an inspiration for future research.

## 1 Introduction

This article provides an overview of evolutionary robotics research where evolution takes place in a population of robots in a continuous manner. Ficici et al. (1999) coined the phrase *embodied evolution* for evolutionary processes that are distributed over the robots in the population to allow them to adapt autonomously and continuously. As robotics technology becomes simultaneously more capable and economically viable, individual robots operated at large expense by teams of experts are increasingly supplemented by collectives of robots used cooperatively under minimal human supervision (Bellingham and Rajan, 2007), and embodied evolution can play a crucial role in enabling autonomous online adaptivity in such robot collectives.

The vision behind embodied evolution is one of the collectives of truly autonomous robots that can adapt their behavior to suit varying tasks and circumstances. Autonomy occurs at two levels: not only the robots perform their tasks without external control but also they assess and adapt—through evolution—their behavior without referral to external oversight and so learn autonomously. This adaptive capability allows robots to be deployed in situations that cannot be accurately modeled *a priori*. This may be because the environment or user requirements are not fully known, or it may be due to the complexity of the interactions among the robots as well as with their environment effectively rendering the scenario unpredictable. Also, onboard adaptivity intrinsically avoids the reality gap (Jakobi et al., 1995) that results from inaccurate modeling of robots or their environment when developing controllers before deployment because controllers continue to develop *after* deployment. A final benefit is that embodied evolution can be seen as parallelizing the evolutionary process because it distributes the evaluations over multiple robots. Alba (2002) has shown that such parallelism can provide substantial benefits, including superlinear speedups. In the case of robots, this has the added benefit of reducing the amount of time spent executing poor controllers per robot, reducing wear and tear.

Embodied evolution’s online nature contrasts with “traditional” evolutionary robotics research. Traditional evolutionary robotics employs evolution in the classical sequential centralized optimization paradigm: parent and survivor selection are centralized and consider the entire population. The “robotics” part entails a series of robotic trials (simulated or not) in an evolution-based search for optimal robot controllers (Nolfi and Floreano, 2000; Bongard, 2013; Doncieux et al., 2015). In terms of task performance, embodied evolution has been shown to outperform alternative evolutionary robotic techniques in some setups such as surveillance and self-localization with flying UAVs (Schut et al., 2009; Prieto et al., 2016), especially regarding convergence speed.

To provide a basis for a clear discussion, we define embodied evolution as a paradigm where evolution is implemented in multirobotic (two or more robots) system. Two robots are already considered a multirobotic system since it is still possible to distribute an algorithm among them. These systems exhibit the following features.

**Decentralized:** There is no central authority that selects parents to produce offspring or individuals to be replaced. Instead, robots assess their performance, exchange, and select genetic material autonomously on the basis of locally available information.

**Online:** Robot controllers change on the fly, as the robots go about their proper actions: evolution occurs during the operational lifetime of the robots and in the robots’ task environment. The process continues after the robots have been deployed.

**Parallel:** Whether they collaborate in their tasks or not, the population consists of multiple robots that perform their actions and evolve concurrently, in the same environment, interacting frequently to exchange genetic material.

The decentralized nature of communicating genetic material implies that the selection is executed locally, usually involving only a part of the whole population (Eiben et al., 2007), and that it must be performed by the robots themselves. This adds a third opportunity for selection in addition to parent and survivor selection as defined for classical evolutionary computing. Thus, embodied evolution extends the collection of operators that define an evolutionary algorithm (i.e., evaluation, selection, variation, and replacement (Eiben and Smith, 2008)) with *mating* as a key evolutionary operator.

**Mating:** An action where two (or more) robots decide to send and/or receive genetic material, whether this material will or will not be used for generating new offspring. When and how this happens depends not only on predefined heuristics but also on evolved behavior, the latter determining to a large extent whether robots ever meet to have the opportunity to exchange genetic material.

In the past 20 years, online evolutionary robotics in general and embodied evolution in particular have matured as research fields. This is evidenced by the growing number of relevant publications in respected evolutionary computing venues such as in conferences (e.g., ACM GECCO, ALIFE, ECAL, and EvoApplications), journals (e.g., Evolutionary Intelligence’s special issue on Evolutionary Robotics (Haasdijk et al., 2014b)), workshops (PPSN 2014 ER workshop, GECCO 2015 and 2017 Evolving collective behaviors in robotics workshop), and tutorials (ALIFE 2014, GECCO 2015 and 2017, ECAL 2015, PPSN 2016, and ICDL-EPIROB 2016). A Google Scholar search of publications citing the seminal embodied evolution paper by Watson et al. (2002) illustrates this growing trend. Since 2009, the paper has attracted substantial interest, more than doubling the yearly number of citations since 2008 (approximately 20 citations per year since then).^1^

To date, however, a clear definition of what embodied evolution is (and what it is not) and an overview of the state of the art in this area are not available. This article provides a definition of the embodied evolution paradigm and relates it to other evolutionary and swarm robotics research (Sections 2 and 3). We identify and review relevant research, highlighting many design choices and issues that are particular to the embodied evolution paradigm (Sections 4 and 5). Together this provides a thorough overview of the relevant state-of-the-art and a starting point for researchers interested in evolutionary methods for collective autonomous adaptation. Section 6 identifies open issues and research in other fields that may provide solutions, suggests directions for future work, and discusses potential applications.

## 2 Context

Embodied evolution considers collectives of robots that adapt online. This section positions embodied evolution vis à vis other methods for developing controllers for robot collectives and for achieving online adaptation.

### 2.1 Offline Design of Behaviors in Collective Robotics

Decentralized decision-making is a central theme in collective robotics research: when the robot collective cannot be centrally controlled, the individual robots’ behavior must be carefully designed so that global coordination occurs through local interactions.

Seminal works from the 1990s such as Mataric’s Nerd Herd (Mataric, 1994) addressed this problem by hand-crafting behavior-based control architectures. Manually designing robot behaviors has since been extended with elaborate methodologies and architectures for multirobot control (see Parker (2008) for a review) and with a plethora of bioinspired control rules for swarm-like collective robotics (see Nouyan et al. (2009) and Rubenstein et al. (2014) for recent examples involving real robots and Beni (2005), Brambilla et al. (2012), and Bayindir (2016) for discussions and recent reviews).

Automated design methods have been explored with the hope of tackling problems of greater complexity. Early examples of this approach were applied to the robocup challenge for learning coordination strategies in a well-defined setting. See the study by Stone and Veloso (1998) for an early review and Stone et al. (2005) and Barrett et al. (2016) for more recent work in this vein. However, Bernstein et al. (2002) demonstrated that solving even the simplest multiagent learning problem is intractable in polynomial time (actually, it is NEXP-complete), so obtaining an optimal solution in reasonable time is currently infeasible. Recent works in reinforcement learning have developed theoretical tools to break down complexity by operating a move from considering many agents to a collection of single agents, each of which being optimized separately (Dibangoye et al., 2015), leading to theoretically well-founded contributions, but with limited practical validation involving very few robots and simple tasks (Amato et al., 2015).

Lacking theoretical foundations, but instead based on the experimental validation, swarm robotics controllers have been developed with black-box optimization methods ranging from brute-force optimization using a simplified (hence tractable) representation of a problem (Werfel et al., 2014) and evolutionary robotics (Hauert et al., 2008; Trianni et al., 2008; Gauci et al., 2012; Silva et al., 2016).

The methods vary, but all the approaches described here (including “standard” evolutionary robotics) share a common goal: to design or optimize a set of control rules for autonomous robots that are part of a collective *before* the actual deployment of the robots. The particular challenge in this kind of work is to design individual behaviors that lead to some required global (“emergent”) behavior without the need for central oversight.

### 2.2 Lifelong Learning in Evolutionary Robotics

It has long been argued that deploying robots in the real world may benefit from continuing to acquire new capabilities *after* initial deployment (Thrun and Mitchell, 1995; Nelson and Grant, 2006), especially if the environment is not known beforehand. Therefore, the question we are concerned with in this article is *how to endow a collective robotics system with the capability to perform lifelong learning*. Evolutionary robotics research into this question typically focuses on individual autonomous robots. Early works in evolutionary robotics that considered lifelong learning explored learning mechanisms to cope with minor environmental changes (see the classic book by Nolfi and Floreano (2000) and Urzelai and Floreano (2001) and (Tonelli and Mouret, 2013) for examples and Mouret and Tonelli (2015) for a nomenclature). More recently, Bongard et al. (2006) and Cully et al. (2015) addressed *resilience* by introducing fast online re-optimization to recover from hardware damage.

Bredeche et al. (2009), Christensen et al. (2010), and Silva et al. (2012) are some examples of online versions of evolutionary robotics algorithms that target the fully autonomous acquisition of behavior to achieve some predefined task in individual robots. Targeting agents in a video game rather than robots, Stanley et al. (2005) tackled the online evolution of controllers in a multiagent system. Because the agents were virtual, the researchers could control some aspects of the evaluation conditions (e.g., restarting the evaluation of agents from the same initial position). This kind of control is typically not feasible in autonomously deployed robotic systems.

Embodied evolution builds on evolutionary robotics to implement lifelong learning in robot *collectives*. Its clear link with traditional evolutionary robotics is exemplified by work such as by Usui and Arita (2003), where a traditional evolutionary algorithm is encapsulated on each robot. Individual controllers are evaluated sequentially in a standard time sharing setup, and the robots implement a communication scheme that resembles an island model to exchange genomes from one robot to another. It is this communication that makes this an instance of embodied evolution.

## 3 Algorithmic Description

This section presents a formal description of the embodied evolution paradigm by means of generic pseudocode and a discussion about its operation from a more conceptual perspective.

The pseudocode in Algorithm 1 provides an idealized description of a robot’s control loop as it pertains to embodied evolution. Each robot runs its own instance of the algorithm, and the evolutionary process emerges from the interaction between the robots. In embodied evolution, there is no entity outside the robots that oversees the evolutionary process, and there is typically no synchronization between the robots: the replacement of genomes is asynchronous and autonomous.

**Algorithm 1:** An individual robot’s control loop for embodied evolution.

initialize robot;	
**for** *ever* **do**	
Sense - Act cycle (depends on robotic paradigm);	
perf ← calculate performance;	
**if** *mating?* **then**	//E.g., is another robot nearby?
transmit my genome;	//and optional further information
*g* ← receive mate’s genome;	
store(*g*);	
**end**	
**if** *replacement?* **then**	//E.g., time or virtual energy runs out
parents ← select parents;	
offspring ← variation(parents);	
activate(offspring)//Time-sharing: control is handed over to the new candidate controller	
**end**	
**end**	

Some steps in this generic control loop can be implicit or entwined in particular implementations. For instance, robots may continually broadcast genetic material over short range, so that other robots that come within this range receive it automatically. In such a case, the *mating* operation is implicitly defined by the selected broadcast range. Similarly, genetic material may be incorporated into the currently active genome as it is received, merging the mating and replacement operations. Implicitly defined or otherwise, the steps in this algorithm are, with the possible exception of performance calculation, necessary components of any embodied evolution implementation.

The following list describes and discusses the steps in the algorithm in detail.

**Initialization:** The robot controllers are typically initialized randomly, but it is possible that the initial controllers are developed offline, be it through evolution or handcraft (e.g., see the work by Hettiarachchi et al. (2006)).

**Sense–act cycle:** This represents “regular,” i.e., not related to the evolutionary process, robot control. The details of the sense–act cycle depend on the robotic paradigm that governs robot behavior; this may include planning, subsumption, or other paradigms. This may also be implemented as a separate parallel process.

**Calculate performance:** If the evolutionary process defines an objective function, the robots monitor their own performance. This may involve measurements of quantities such as speed, number of collisions, or amount of collected resources. Whatever their nature, these measurements are then used to evaluate and compare genomes (as fitness values in evolutionary computation). The possible discrepancy between the individual’s objective function and the population welfare will be discussed further in Section 6.2.

**Mating:** This is the essential step in the evolutionary process where robots exchange genetic material. The choice to mate with another robot may be purely based on the environmental contingencies (e.g., when robots mate whenever they are within communication range), but other considerations may also play a part (e.g., performance, genotypic similarity). The pseudocode describes a symmetric exchange of genomes (both with a transmit and a receive operation), but this may be asymmetrical for particular implementations. In implementations such as that of Schwarzer et al. (2011) or Haasdijk et al. (2014a), for instance, robots suspend normal operation to collect genetic material from other, active robots. Mating typically results in a pool of candidate parents that are considered in the parent selection process.

**Replacement:** The currently active genome is replaced by a new individual (the offspring), implying the removal of the current genome. This event can be triggered by a robot’s internal conditions (e.g., running out of time or virtual energy, reaching a given performance level) or through interactions with other robots (e.g., receiving promising genetic material (Watson et al., 2002)).

**Parent selection:** This is the process that selects which genetic information will be used for the creation of new offspring from the received genetic information through mating events. When an objective is defined, the performance of the received genome is usually the basis for selection, just as in regular evolutionary computing. In other cases, the selection among received genomes can be random or depend on non-performance related heuristics (e.g., random, genotypic proximity). In the absence of objective-driven selection pressure, individuals are still competing with respect to their ability to spread their own genome within the population, although that cannot be explicitly captured during parent selection. This will be further discussed in Section 5.2.

**Variation:** A new genome is created by applying the variation operators (mutation and crossover) on the selected parent genome(s). This is subsequently activated to replace the current controller.

**Figure 1:**
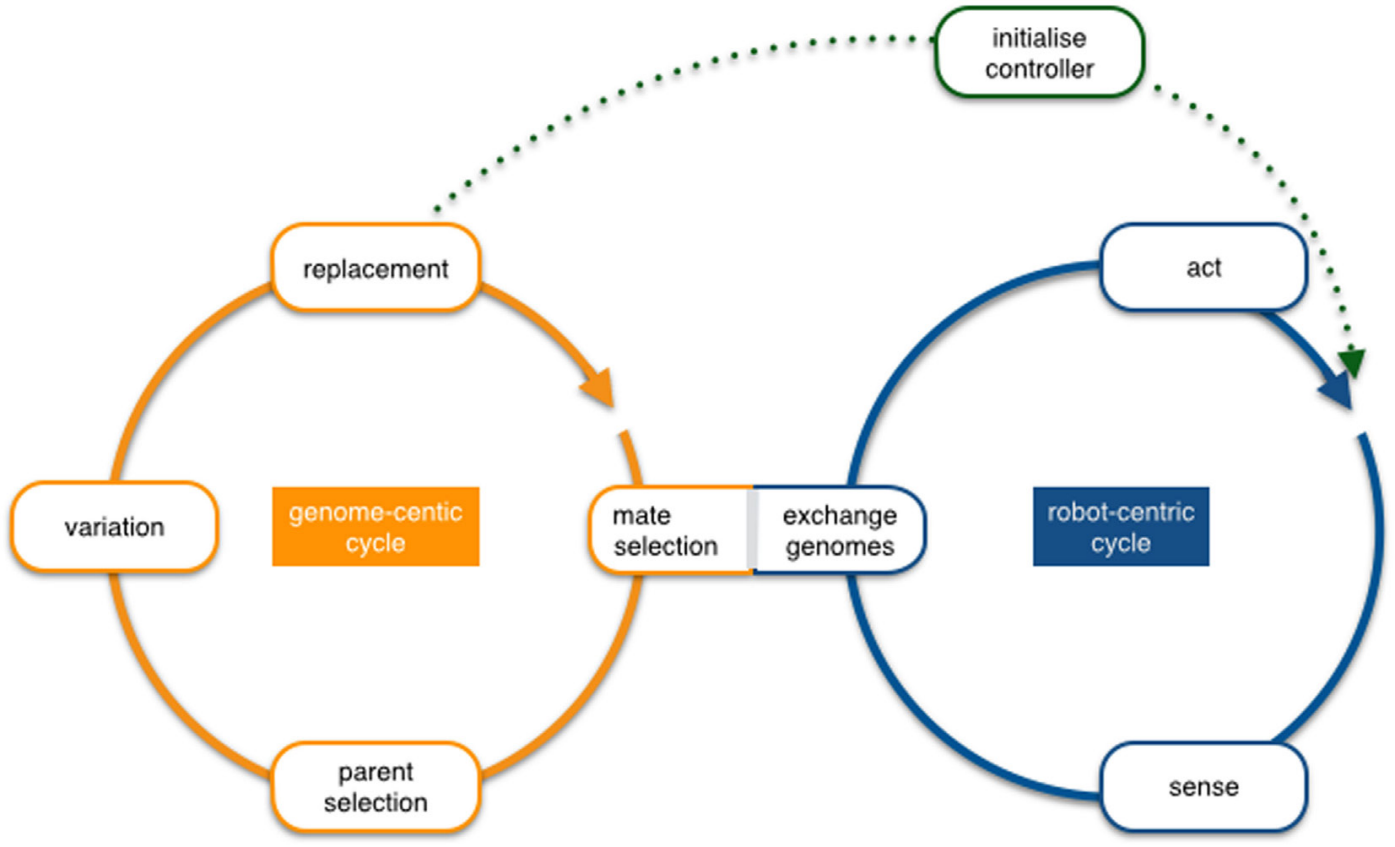
The overlapping robot-centric and genome-centric cycles in embodied evolution. The robot-centric cycle uses a single *active* genome that determines the current robot behavior (sense–act loop), and the genome-centric cycle manages an internal *reservoir* of genomes received from other robots or built locally (parent selection/variation), of which the next active genome will be selected eventually (replacement).

From a conceptual perspective, embodied evolution can be analyzed at two levels that are represented by two as depicted in Figure 1.

**The robot-centric cycle** is depicted on the right in Figure 1. It represents the physical interactions that occur between the robot and its environment, including interactions with other robots and extends this sense–act loop commonly used to describe real-time control systems by accommodating the exchange and activation of genetic material. At this particular point, the genome-centric and robot-centric cycles overlap. The cycle operates as follows: each robot is associated with an *active* genome, and the genome is interpreted into a set of features and control architecture (the phenotype), which produces a behavior that includes the transmission of its own genome to some other robots. Each robot eventually switches from an active genome to another, depending on a specific event (e.g., minimum energy threshold) or duration (e.g., fixed lifetime), and consequently changes its active genome, probably impacting its behavior.

**The genome-centric cycle** deals with the events that directly affect the genomes existing in the robot population and therefore also the evolution *per se*. Again, the mating and the replacement are the events that overlap with the robot-centric cycle. The operation from the genome cycle perspective is as follows: each robot starts with an initial genome, either initialized randomly or *a priori* defined. While this genome is *active*, it determines the phenotype of the robot, hence its behavior. Afterward, when replacement is triggered, some genomes are selected from the reservoir of genomes previously received according to the parent selection criteria and later combined using the variation operators. This new genome will then become part of the population. In the case of fixed-size population algorithms, the replacement will automatically trigger the removal of the old genome. In some other cases, however, there is a specific criterion to trigger the removal event producing populations of individuals that change their size along the evolution.

The two circles connect on two occasions, first by the “exchange genomes” (or mating) process, which implies the transmission of genetic material, possibly together with additional information (fitness if available, general performance, genetic affinity, etc.) to modulate future selection. Generally, the received information is stored to be used (in full or in part) to replace the active genome in the later parent selection process. Therefore, the event is triggered and modulated by the robot cycle, but it impacts on the genomic cycle. Also, the decentralized nature of the paradigm enforces that these transmissions occur locally, either one-to-one or to any robot in a limited range. There are several ways in which mate selection can be implemented, for instance, individuals may send and receive genomic information indiscriminately within a certain location range or the frequency of transmission can depend on the task performance. The second overlap between the two cycles is the activation of new genomic information (replacement). The activation of a genome in the genomic cycle implies that this new genome will now take control of the robot and therefore changes the response of the robot in the scenario (in evolutionary computing terms, this event will mark the start of a new individual evaluation). This aspect is what creates the online character of the algorithm that, together with the locality constraints, implies that the process is also asynchronous.

This conceptual representation matches what has been defined as *distributed* embodied evolution by Eiben et al. (2010). The authors proposed a taxonomy for online evolution that differentiates between encapsulated, distributed, and hybrid schemes. Most embodied evolution implementations are distributed, but this schematic representation also covers hybrid implementations. In such cases, the robot locally maintains a population that is augmented through mating (rather like an island model in parallel evolutionary algorithms). It should be noted that encapsulated implementations (where each robot runs independently of the others) are not considered in this overview.

## 4 Embodied Evolution: The State of the Art

In this section, we identify the major research topics from the works published since the inception of the domain, all summarized in Table 1. Table 1 provides an overview of published research on embodied evolution with robot collectives. Each entry describes a contribution, which may cover several papers. The entries are described in terms of their implementation details, the robot behavior, experimental settings, mating conditions, selection, and replacement schemes. The glossary in Table 2 explains these features in more detail.

**Table 1:** Overview of Embodied Evolution research.

	Implementation				Robot behavior					Experimental settings		Mating conditions			Selection scheme			Replacement scheme			
					
	Distributed	Hybrid	Simulation	Real robots	Monomorphic	Polymorphic	Individual behavior	Cooperation	Division of labor	Task	No of robots	Panmictic	Proximity	Other	Performance	Random	Genotypic distance	Fixed lifetime	Variable lifetime	Event based	Limited lifetime
Ficici et al. (1999); Watson et al. (2002)	•			•	•		•			Phototaxis	8		•		•				•		

Simões and Dimond (2001)	•			•	•		•			Obstacle avoidance	6	•			•			•			

Usui and Arita (2003)		•		•	•		•			obstacle avoidance	6		•		•			•			

Bianco and Nolfi (2004)	•		•		•		•			Self-assembly	64		•			•				•	

Hettiarachchi et al. (2006); Hettiarachchi and Spears (2009)	•		•		•		•			Navigation with obstacle avoidance	60		•		•					•	

Wischmann et al. (2007)	•		•		•		•			Foraging^a^	3		•		•				•		

Perez et al. (2008)		•	•		•		•			Obstacle avoidance	5	•			•			•			

König and Schmeck (2009); König et al. (2009)	•		•		•		•			Obstacle avoidance with gate passing	26; 30		•		•			•			

Pugh and Martinoli (2009)		•	•	•	•		•			Obstacle avoidance	1–10	•	•		•			•			

Prieto et al. (2009); Trueba et al. (2011, 2012)	•		•			•		•	•	Surveillance, foraging, construction	20		•		•					•	•

Bredeche and Montanier (2010, 2012) Bredeche (2014)	•		•	•	•			•		None	20; 4000		•			•					•

Prieto et al. (2010)	•			•		•		•	•	Surveillance	8		•		•					•	•

Schwarzer et al. (2010)	•		•	•		•		•		None	Up to 40		•			•			•		

Schwarzer et al. (2011)	•		•		•		•			Phototaxis	4		•		•			•			

Montanier and Bredeche (2011, 2013)	•		•		•			•		None	100		•			•	•				•

Huijsman et al. (2011)	•	•	•		•		•			Obstacle avoidance	4–400	•		•	•			•			

Karafotias et al. (2011)		•	•		•		•		•	Obstacle avoidance, phototaxis, and patrolling	10			•	•			•			

Silva et al. (2012, 2013, 2015, 2017)		•	•	•		•	•	•		Navigation, aggregation, surveillance, and phototaxis	2–20		•		•					•	

Weel et al. (2012a,b)		•	•		•			•		Foraging	10; 50		•		•			•			

García-Sánchez et al. (2012)		•	•		•		•			Obstacle avoidance	4–36		•	•	•			•			

Haasdijk and Bredeche (2013) Haasdijk et al. (2013, 2014a); Noskov et al. (2013); Haasdijk and Eigenhuis (2016); Bangel and Haasdijk (2017); Kemeling and Haasdijk (2017)	•		•			•	•		•	Foraging	100		•		•			•			

Trueba et al. (2013) Trueba (2017)	•		•	•		•		•	•	Synthetic mapping, gathering, self-location	40; 20; 9	•	•		•	•				•	•

O’Dowd et al. (2014)		•		•	•		•			Foraging	10		•		•			•			

Fernandez Pérez et al. (2014)	•		•		•				•	Foraging	50		•		•	•		•			

Fernandez Pérez et al. (2015)	•		•		•			•		Foraging	100		•		•					•	

Hart et al. (2015); Steyven et al. (2016)	•		•		•			•		Foraging	100		•		•						•

Heinerman et al. (2015, 2016)		•	•	•	•		•			Obstacle avoidance	6	•			•			•			

Montanier et al. (2016); Bredeche et al. (2017)	•		•			•			•	Foraging	100; 500		•		•	•					•

Fernandez Pérez et al. (2017)	•		•			•		•		Foraging	200		•		•			•			

*^a^As a proxy for predator avoidance*.

**Table 2:** Glossary.

Field	Comment
Implementation	**Distributed** implementations have one genome for each robot, and an offspring is created only as the result of a mating event or by mutating the current genome. **Hybrid** implementations have multiple genomes per robot, and offspring can be created from this internal pool and from genomes “imported” through mating events. As stated earlier, the encapsulated scheme is not considered embodied evolution as there is no exchange of genomes between robots in this case.
	The experiments can use **real robots** or **simulation**.

Robot behavior	A **monomorphic** population contains individuals with similar genotypes (with variations due to mutation). A **polymorphic** population is divided into two (or more) subgroups of genetically similar individuals, and different genotypic signatures from one group to the other, e.g., to achieve specialization.
	We distinguish between experiments that target efficient **individual behavior** vs. collective behaviors (i.e., social behaviors, incl. cooperation)

Experimental settings	Identifies the **task(s)** considered in the experiment, e.g., obstacle avoidance, foraging, … **None** indicates that there is no user-defined task and that consequently, selection pressure results from the environment only. The **number of robots** used is also included. *n*_1_ − *n*_2_ indicates the interval for one experiment and *n*1; *n*2 gives numbers for two experiments.

Mating conditions	Mating can be based on **proximity**: two robots can mate whenever they are physically close to each other (e.g., in infrared communication range). In **panmictic** systems, robots can mate with all other robots, regardless of their location. **Other** comprises systems where robots maintain an explicit list of potential mates (a social network), which may be maintained through gossiping.

Selection scheme	Parents are selected from the received and internal genomes on the basis of their **performance** if a task is defined. **Random** parent selection implies only environment-driven selection. Currently, the only examples of other selection schemes use genotypic distance, but this category also covers metrics such as novelty.

Replacement scheme	Genomes can have a **fixed lifetime**, **variable lifetime**, or **limited lifetime** (similar to variable lifetime, but with an upper bound). **Event-based** replacement schemes do not depend on time but on events such as reception of genetic material (e.g., in the microbial GA used by Watson et al. (2002)).

First, we distinguish between works that consider embodied evolution as a parallel search method for optimizing *individual* behaviors and works where embodied evolution is employed to craft *collective* behavior in robot populations. The latter trend, where the emphasis is on collective behavior, has emerged relatively recently and since then has gained importance (32 papers since 2009).

Second, we consider the homogeneity of the evolving population; borrowing definitions from biology, we use the term *monomorphic* (resp. *polymorphic*) for a population containing one (resp. more than one) class of genotype, for instance, to achieve specialization. A monomorphic population implies that individuals will behave in a similar manner (except for small variations due to minor genetic differences). On the contrary, polymorphic populations host multiple groups of individuals, each group with its particular genotypic signature, possibly displaying a specific behavior. Research to date shows that cooperation in monomorphic populations can be easily achieved (e.g. (Prieto et al., 2010; Schwarzer et al., 2010; Montanier and Bredeche, 2011, 2013; Silva et al., 2012)), while polymorphic populations (e.g., displaying genetic-encoded behavioral specialization) require very specific conditions to evolve (e.g., Trueba et al. (2013); Haasdijk et al. (2014a); Montanier et al. (2016)).

A notable number of contributions employ real robots. Since the first experiments in this field, the intrinsic online nature of embodied evolution has made such validation comparatively straightforward (Ficici et al., 1999; Watson et al., 2002). “Traditional” evolutionary robotics is more concerned with robustness at the level of the evolved *behavior* (mostly caused from the reality gap that exists between simulation and the real world) than is embodied evolution, which emphasizes the design of robust *algorithms*, where transfer between simulation and real world may be less problematic. In the contributions presented here, simulation is used for extensive analysis that could hardly take place with real robots due to time or economic constraint. Still, it is important to note that many researchers who use simulation have also published works with real robots, thus including real-world validation in their research methodology.

Since 2010, there have been a number of experiments that employ large (≥100) numbers of (simulated) robots, shifting toward more swarm-like robotics where evolutionary dynamics can be quite different (Huijsman et al., 2011; Bredeche, 2014; Haasdijk et al., 2014b). Recent works in this vein focus on the nature of selection pressure, emphasizing the unique aspect of embodied evolution that selection pressure results from both the environment (which impacts mating) and the task. Bredeche and Montanier (2010, 2012) showed that environmental pressure alone can drive evolution toward self-sustaining behaviors. Haasdijk et al. (2014a) showed that these selection pressures can to some extent be modulated by tuning the ease with which robots can transmit genomes. Steyven et al. (2016) showed that adjusting the availability and value of energy resources results in the evolution of a range of different behaviors. These results emphasize that tailoring the environmental requirements to transmit genomes can profoundly impact the evolutionary dynamics and that understanding these effects is vital to effectively develop embodied evolution systems.

## 5 Issues in Embodied Evolution

What sets embodied evolution apart from classical evolutionary robotics (and, indeed, from most evolutionary computing) is the fact that evolution acts as a force for continuous adaptation, not (just) as an optimizer before deployment. As a continuous evolutionary process, embodied evolution is similar to some evolutionary systems considered in artificial life research (e.g. Axelrod (1984); Ray (1993), to name a few). The operations that implement the evolutionary process to adapt the robots’ controllers are an integral part of their behavior in their task environment. This includes mating behavior to exchange and select genetic material, assessing one’s own and/or each other’s task performance (if a task is defined) and applying variation operators such as mutation and recombination.

This raises issues that are particular to embodied evolution. The research listed in the previous section has identified and investigated a number of these issues, and the remainder of this section discusses these issues in detail, while Section 6.2 discusses issues that so far have not benefited from close attention in embodied evolution research.

### 5.1 Local Selection

In embodied evolution, the evolutionary process is generally implemented through local interactions between the robots, i.e., the mating operation introduced above. This implies the concept of a neighborhood from which mates are selected. One common way to define neighborhood is to consider robots within communication range, but it can also be defined in terms of other distance measures such as genotypic or phenotypic distance. Mates are selected by sampling from this neighborhood, and a new individual is created by applying variation operators to the sampled genome(s). This local interaction has its origin in constraints that derive from communication limitations in some distributed robotic scenarios. Schut et al. (2009) showed it to be beneficial in simulated setups as an exploration/exploitation balancing mechanism.

Embodied evolution, with chance encounters providing the sampling mechanism, has some similarities with other flavors of evolutionary computation. Cellular evolutionary algorithms (Alba and Dorronsoro, 2008) consider continuous random rewiring of a network topology (in a grid of CPUs or computers) where all elements are evaluated in parallel. In this context, locally selecting candidates for reproduction is a recurring theme that is shared with embodied evolution (e.g., García-Sánchez et al. (2012); Fernandez Pérez et al. (2014)).

### 5.2 Objective Functions vs Selection Pressure

In traditional evolutionary algorithms, the optimization process is guided by a (set of) objective function(s) (Eiben and Smith, 2008). Evaluation of the candidate solutions, i.e., of the genomes in the population, allows for (typically numerical) comparison of their performance. Beyond its relevance for performance assessment, the evaluation process *per se* has generally no influence on the manner in which selection, variation, and replacement evolutionary operators are applied. This is different in embodied evolution, where the behavior of an individual can directly impact the likelihood of encounters with others and so influence selection and reproductive success (Bredeche and Montanier, 2010). Evolution can not only improve task performance but can also develop mating strategies, for example, by maximizing the number of encounters between robots if that improves the likelihood of transmitting genetic material.

It is therefore important to realize that the *selection pressure* on the robot population does not only derive from the specified *objective function(s)* as it traditionally does in evolutionary computation. In embodied evolution, the environment, including the mechanisms that allow mating, also exert selection pressure. Consequently, evolution experiences selection pressure from the aggregate of objective function(s) and environmental particularities. Steyven et al. (2016) researched how aspects of the robots’ environment influence the emergence of particular behaviors and the balance between pressure toward survival and task. The objective may even pose requirements that are opposed to those by the environment. This can be the case when a task implies risky behaviors or because a task requires resources that are also needed for survival and mating. In such situations, the evolutionary process must establish a tradeoff between objective-driven optimization and the maintenance of a viable environment where evolution occurs, which is a challenge in itself (Haasdijk, 2015).

### 5.3 Autonomous Performance Evaluation

The decentralized nature of the evolutionary process implies that there is no omniscient presence who knows (let alone determines) the fitness values of all individuals. Consequently, when an objective function is defined, it is the robots themselves that must gage their performance and share it with other robots when mating: each robot must have an evaluation function that can be computed onboard and autonomously. Typical examples of such evaluation functions are the number of resources collected, the number of times a target has been reached, or the number of collisions. The requirement of autonomous assessment does not fundamentally change the way one defines fitness functions, but it does impact their usage as shown by Nordin and Banzhaf (1997), Walker et al. (2006), and Wolpert and Tumer (2008).

**Evaluation time:** The robots must run a controller for some time to assess the resultant behavior. This implies a *time sharing* scheme where robots run their current controllers to evaluate their performance. In many similar implementations, a robot runs a controller for a fixed evaluation time; Haasdijk et al. (2012) showed that this is a very important parameter in encapsulated online evolution, and it is likely to be similarly influential in embodied evolution.

**Evaluation in varying circumstances:** Because the evolutionary machinery (mating, evaluating new individuals, etc.) is an integral part of robot behavior, which runs in parallel with the performance of regular tasks, there can be no thorough re-initialization or re-positioning procedure between genome replacements. This implies a noisy evaluation: a robot may undervalue a genome starting in adverse circumstances and vice versa. As Nordin and Banzhaf (1997) (p. 121) put it: *“Each individual is tested against a different real-time situation leading to a unique fitness case. This results in ‘unfair’ comparison where individuals have to navigate in situations with very different possible outcomes. However, [*…*] experiments show that over time averaging tendencies of this learning method will even out the random effects of probabilistic sampling and a set of good solutions will survive*.*”* Bredeche et al. (2009) proposed a re-evaluation scheme to address this issue: seemingly efficient candidate solutions have a probability to be re-evaluated to cope with possible evaluation noise. A solution with a higher score *and* a lower variance will then be preferred to one with a higher variance. While re-evaluation is not always used in embodied evolution, the evaluation of relatively similar genomes on different robots running in parallel provides another way to smooth the effect of noisy evaluations.

**Multiple objectives:** To deal with multiple objectives, evolutionary computation techniques typically select individuals on the basis of Pareto dominance. While this is eminently possible when selecting partners as well, Pareto dominance can only be determined vis à vis the population sample that the selecting robot has acquired. It is unclear how this affects the overall performance and if the robot collective can effectively cover the Pareto front. Bangel and Haasdijk (2017) investigated the use of a “market mechanism” to balance the selection pressure over multiple tasks in a concurrent foraging scenario, showing that this at least prevents the robot collective from focusing on single tasks, but that it does not lead to specialization in individual robots.

## 6 Discussion

The previous sections show that there is a considerable and increasing amount of research into embodied evolution, addressing issues that are particular to its autonomous and distributed nature. This section turns to the future of embodied evolution research, discussing potential applications and proposing a research agenda to tackle some of the more relevant and immediate issues that so far have remained insufficiently addressed in the field.

### 6.1 Applications of Embodied Evolution

Embodied evolution can be used as a design method for engineering, as a modeling method for evolutionary biology, or as a method to investigate evolving complex systems more generally. Let us briefly consider each of these possibilities.

**Engineering:** The online adaptivity afforded by embodied evolution offers many novel possibilities for deployment of robot collectives: exploration of unknown environments, search and rescue, distributed monitoring of large objects or areas, distributed construction, distributed mining, etc. Embodied evolution can offer a solution when robot collectives are required to be versatile, since the robots can be deployed in and adapt to open and *a priori* unknown environments and tasks. The collective is comparatively robust to failure through redundancy and the decentralized nature of the algorithm because the system continues to function even if some robots break down. Embodied evolution can increase autonomy because the robots can, for instance, learn how to maintain energy while performing their task without intervention by an operator.

Currently, embodied evolution has already provided solutions to tasks such as navigation, surveillance, and foraging (see Table 1 for a complete list), but these are of limited interest because of the simplicity of the tasks considered in research to date. The research agenda proposed in Section 6.2 provides some suggestions for further research to mitigate this.

**Evolutionary biology:** In the past 100 years, evolutionary biology benefited from both experimental and theoretical advances. It is now possible, for instance, to study evolutionary mechanisms through methods such as gene sequencing (Blount et al., 2012; Wiser et al., 2013). However, *in vitro* experimental evolution has its limitations: with evolution in “real” substrates, the time scales involved limit the applicability to relatively simple organisms such as *Escherichia coli* (Good et al., 2017). From a theoretical point of view, population genetics (see Charlesworth and Charlesworth (2010) for a recent introduction) provides a set of mathematically grounded tools for understanding evolution dynamics, at the cost of many simplifying assumptions.

Evolutionary robotics has recently gained relevance as an individual-based modeling and simulation method in evolutionary biology (Floreano and Keller, 2010; Waibel et al., 2011; Long, 2012; Mitri et al., 2013; Ferrante et al., 2015; Bernard et al., 2016), enabling the study of evolution in populations of robotic individuals in the physical world. Embodied evolution enables more accurate models of evolution because it is possible to embody not only the physical interactions but also the evolutionary operators themselves.

**Synthetic approach:** Embodied evolution can also be used to “understand by design” (Pfeifer and Scheier, 2001). As Maynard Smith (1992), a prominent researcher in evolutionary biology, advocated in a famous (Maynard Smith, 1992)’s Science paper (originally referring to Tierra (Ray, 1993)): “so far, we have been able to study only one evolving system and we cannot wait for interstellar flight to provide us with a second. If we want to discover generalizations about evolving systems, we have to look at artificial ones.”

This *synthetic approach* stands somewhere between biology and engineering, using tools from the latter to understand mechanisms originally observed in nature and aiming at identifying general principles not confined to any particular (biological) substrate. Beyond improving our *understanding* of adaptive mechanisms, these general principles can also be used to improve our ability to *design* complex systems.

### 6.2 Research Agenda

We identify a number of open issues that need to be addressed so that embodied evolution can develop into a relevant technique to enable online adaptivity of robot collectives. Some of these issues have been researched in other fields (e.g., credit assignment is a well-known and often considered topic in reinforcement learning research). Lessons can and should be learned from there, inspiring embodied evolution research into the relevance and applicability of findings in those other fields.

In particular, we identify the following challenges.

**Benchmarks:** The pseudocode in Section 3 provides a clarification of embodied evolution’s concepts by describing the basic building blocks of the algorithm. This is only a first step toward a theoretical and practical framework for embodied evolution. Some authors have already taken steps in this direction. For instance, Prieto et al. (2015) propose an abstract algorithmic model to study both general and specific properties of embodied evolution implementations. Montanier et al. (2016) described “vanilla” versions of embodied evolution algorithms that can be used as practical benchmarks. Further exploration of abstract models for theoretical validation is needed. Also, standard benchmarks and test cases, along with systematically making the source code available, would provide a solid basis for empirical validation of individual contributions.

**Evolutionary dynamics:** Embodied evolution requires new tools for analyzing the evolutionary dynamics at work. Because the evolutionary operators apply *in situ*, the dynamics of the evolutionary process are not only important in the context of understanding or improving an optimization procedure, but they also have a direct bearing on how the robots behave and change their behavior when deployed.

Tools and methodologies to characterize the dynamics of evolving systems are available. The field of population genetics has produced techniques for estimating the selection pressure compared to genetic drift possibly occurring in finite-sized populations (see, for instance, Wakeley (2008) and Charlesworth and Charlesworth (2010) for a comprehensive introduction). Similarly, tools from adaptive dynamics (Geritz et al., 1998) can be used to investigate how particular solutions spread within the population. Finally, embodied evolution produces phylogenetic trees that can be studied either from a population genetics viewpoint (e.g., coalescence theory to understand the temporal structure of evolutionary adaptation) and graph theory (e.g., to characterize the particular structure of the inheritance graph). Boumaza (2017) shows an interesting first foray into using this technique to analyze embodied evolution.

**Credit assignment:** In all the research reviewed in this article that considers robot tasks, the fitness function is defined and implemented at the level of the individual robot: it assesses its own performance independently of the others. However, collectively solving a task often requires an assessment of performance at group level rather than individual level. This raises the issue of estimating each individual’s contribution to the group’s performance, which is unlikely to be completely captured by a fitness function (e.g. all individuals going toward the single larger food patch may not always be the best strategy if one aim to bring back the largest amount of food to the nest).

Closely related to our concern, Stone et al. (2010) formulated the *ad hoc teamwork* problem in multirobot systems, involving robots that each must “collaborate with previously unknown teammates on tasks to which they are all individually capable of contributing as team members.” As stated by Wolpert and Tumer (2008), this implies devoting substantial attention to the problem of estimating the *local utility* of individual agents with respect to the *global welfare* of the whole group and how to make a tradeoff between individual and group performance (e.g., Hardin (1968); Arthur (1994)).

While a generally applicable method to estimate an individual’s local utility in an online distributed setting has so far eluded the community, it is possible to provide an exact assessment in controlled settings. Methods from cooperative game theory, such as computing the Shapley value (Shapley, 1953), could be used in embodied evolution but are computationally expensive and require the ability to replay experiments. However, replaying experiments is possible only with simulation and/or controlled experimental settings. While these methods cannot apply when robots are deployed in the real world, they at least provide a method to *compare* the outcome of candidate solutions to estimate individuals’ marginal contributions and choose which should be deployed.

**Social complexity:** Section 4 shows that embodied evolution so far demonstrated only a limited set of social organization concepts: simple cooperative and division of labor behaviors. To address more complex tasks, we must first gain a better understanding of the mechanisms required to achieve complex collective behaviors. This raises two questions. First, there is an *ethological* question: what are the behavioral mechanisms at work in complex collective behaviors? Some of them, such as positive and negative feedback between individuals, or indirect communication through the environment (i.e., *stigmergy*), are well known from examples found both in biology (Camazine et al., 2003) and theoretical physics (Deutsch et al., 2012). Second, there is a question about the origins and stability of behaviors: what are the key elements that make it possible to evolve collective behaviors, and what are their limits? Again, evolutionary ecology provides relevant insights, such as the interplay between the level of cooperation and relatedness between individuals (West et al., 2007). The literature on such phenomena in biological systems may provide a good basis for research into the evolution of social complexity in embodied evolution.

A first step would be to clearly define the nature of social complexity that is to be studied. For this, evolutionary game theory (Maynard Smith, 1992) has already produced a number of well-grounded and well-defined “games” that capture many problems involving interactions among individuals, including thorough analysis of the evolutionary dynamics in simplified setups. Of course, results obtained on abstract models may not be transferable within more realistic settings (as Bernard et al. (2016) showed for mutualistic cooperation), but the systematic use of a formal problem definition would greatly benefit the clarity of contributions in our domain.

**Open-ended adaptation:** As stated in Section 2, embodied evolution aims to provide continuous adaptation so that the robot collective can cope with changes in the objectives and/or the environment. Montanier and Bredeche (2011) showed that embodied evolution enables the population to react appropriately to changes in the regrowth rate of resources, but generally this aspect of embodied evolution has to date not been sufficiently addressed.

We reformulate the goal of continuous adaptation as providing *open-ended* adaptation, i.e., having the ability to continually keep exploring new behavioral patterns, constructing increasingly complex behaviors as required. Bedau et al. (2000), Soros and Stanley (2014), and Taylor et al. (2016) and others identified open-ended adaptation in artificial evolutionary systems as one of the big questions of artificial life. Open-ended adaptation in artificial systems, in particular in combination with learning relevant task behavior, has proved to be an elusive ambition.

A possible avenue to achieve this ambition may lie in the use of quality diversity approaches in embodied evolution. Recent research has considered *quality diversity* measures as a replacement (Lehman and Stanley, 2011) or additional (Mouret and Doncieux, 2012) objective to improve the population diversity and consequently the efficacy of evolution. To date, such research has focused on the evolution of behavior for particular tasks with task-specific metrics of behavioral diversity that must be tailored for each application. To be able to exploit quality diversity in unknown environments and for arbitrary tasks, generic measures of behavioral diversity must be developed.

Another avenue of research would be to take inspiration from the behavior of a passerine bird, the great tit (parus major), as recently analyzed by Aplin et al. (2017). It appears that great tits combine collective and individual learning with varying intensity as they age and that the motivations to pursue behaviors also vary with age. Reward-based learning occurs primarily in young birds and is often individual, while adult birds engage mostly in social learning to copy the behavior that is most common, regardless of whether it produces more or less rewards than alternative behavior. This combination of conformist and payoff-sensitive reinforcement allows individuals and populations both to acquire adaptive behavior and to track environmental change.

Combining embodied evolution, individual reinforcement learning with task-based and diversity-enhancing objectives may yield similar behavioral plasticity for collectives of robots.

**Safety and robot ethics:** To deploy the kind of adaptive technology that embodied evolution aims for responsibly, one must ensure that the adaptivity can be controlled: autonomous adaptation carries the risk of adaptation developing in directions that do not meet the needs of human users or that they even may find undesirable. Even so, the adaptive process should be curtailed as little as possible to allow effective, open-ended, learning. The user cannot be expected to monitor and closely control the robot’s behavior and learning process; this may in fact be impossible in exactly those scenarios where robotic autonomy is most beneficial and adaptivity most urgently required. There is growing awareness that it may be necessary to endow robots with innately ethical behavior (e.g., Moor (2006); Anderson and Anderson (2007); Vanderelst and Winfield (2018)), where the systems select actions based on a “moral arithmetic” (Bentham, 1878), often informed by casuistry, i.e., generalizing morality on the basis of example cases in which there is agreement concerning the correct response (Anderson and Anderson, 2007). Moral reasoning along these lines could conceivably be enabled in embodied evolution as well, in which case interactive evolution to develop surrogate models of user requirements may offer one possible route to allow user guidance.

Additional open issues and opportunities will no doubt arise from advances in this and other fields. A relevant recent development, for instance, is the possibility of evolvable morphofunctional machines that are able to change both their software *and* hardware features (Eiben and Smith, 2015) and replicate through 3D printing (Brodbeck et al., 2015). This would allow embodied evolution holistically to adapt the robots’ morphologies as well as their controllers. This can have profound consequences for embodied evolution implementations that exploit these developments: it would, for instance, enable dynamic population sizes, allowing for more risky behavior as broken robots could be replaced or recycled.

## 7 Conclusion

This article provides an overview of embodied evolution for robot collectives, a research field that has been growing since its inception around the turn of the millennium. The main contribution of this article is threefold. First, it clarifies the definitions and overall process of embodied evolution. Second, it presents an overview of embodied evolution research conducted to date. Third, it provides directions for future researches.

This overview sheds light on the maturity of the field: while embodied evolution was mostly used as a parallel search method for designing individual behavior during its first decade of existence, a trend has emerged toward its collective aspects (i.e., cooperation, division of labor, specialization). This trend goes hand in hand with a trend toward larger, swarm-like, robot collectives.

We hope this overview will provide a stepping stone for the field, accounting for its maturity and acting as an inspiration for aspiring researchers. To this end, we highlighted possible applications and open issues that may drive the field’s research agenda.

## Author Contributions

All authors listed have made a substantial, direct, and intellectual contribution to the work equally and approved it for publication.

## Conflict of Interest Statement

The authors declare that the research was conducted in the absence of any commercial or financial relationships that could be construed as a potential conflict of interest.

## Footnotes

^1^See https://plot.ly/~evertwh/17/ for more details and the underlying data.
